# Structural Insights into Methylated DNA Recognition by the Methyl-CpG Binding Domain of MBD6 from *Arabidopsis thaliana*

**DOI:** 10.1021/acsomega.1c04917

**Published:** 2022-01-19

**Authors:** Yutaka Mahana, Izuru Ohki, Erik Walinda, Daichi Morimoto, Kenji Sugase, Masahiro Shirakawa

**Affiliations:** † Department of Molecular Engineering, Kyoto University, Kyoto-Daigaku Katsura, Nishikyo-Ku, Kyoto 615-8510, Japan; ‡ Institute for Chemical Research, Kyoto University, Gokasho, Uji, Kyoto 611-0011, Japan; § Graduate School of Medicine, Kyoto University, Yoshida Konoe-Cho, Sakyo-Ku, Kyoto 606-8501, Japan

## Abstract

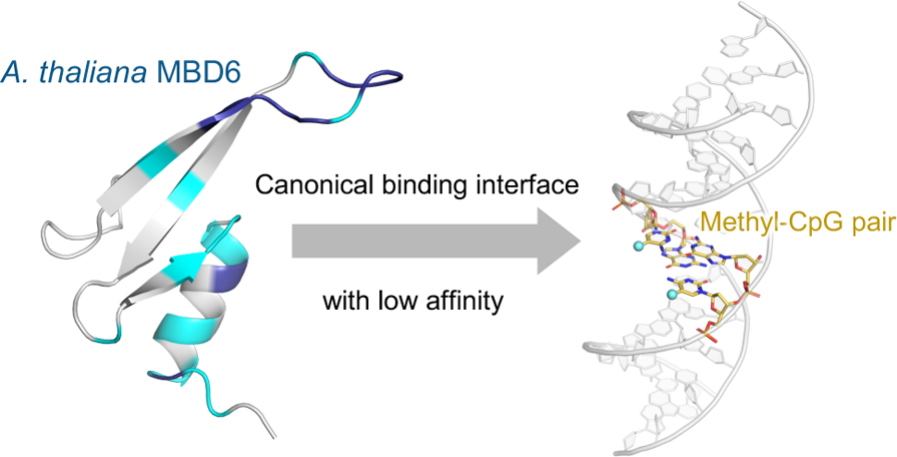

Cytosine methylation is an epigenetic modification essential for formation of mature heterochromatin, gene silencing, and genomic stability. In plants, methylation occurs not only at cytosine bases in CpG but also in CpHpG and CpHpH contexts, where H denotes A, T, or C. Methyl-CpG binding domain (MBD) proteins, which recognize symmetrical methyl-CpG dinucleotides and act as gene repressors in mammalian cells, are also present in plant cells, although their structural and functional properties still remain poorly understood. To fill this gap, in this study, we determined the solution structure of the MBD domain of the MBD6 protein from *Arabidopsis thaliana* and investigated its binding properties to methylated DNA by binding assays and an in-depth NMR spectroscopic analysis. The AtMBD6 MBD domain folds into a canonical MBD structure in line with its binding specificity toward methyl-CpG and possesses a DNA binding interface similar to mammalian MBD domains. Intriguingly, however, the binding affinity of the AtMBD6 MBD domain toward methyl-CpG-containing DNA was found to be much lower than that of known mammalian MBD domains. The main difference arises from the absence of positively charged residues in AtMBD6 that supposedly interact with the DNA backbone as seen in mammalian MBD/methyl-CpG-containing DNA complexes. Taken together, we have established a structural basis for methyl-CpG recognition by AtMBD6 to develop a deeper understanding how MBD proteins work as mediators of epigenetic signals in plant cells.

## Introduction

A certain percentage of cytosine bases in eukaryotic DNA exist in an epigenetically modified form, as 5-methylcytosine. Cytosine methylation plays essential roles in numerous vital functions such as repression of gene expression, organization of the chromatin structure, and inactivation of transposons.^1,2^ In animal cells, cytosine methylation occurs mostly at symmetrical CpG sequences and is achieved by the action of DNA methyltransferases.^1,3^ 5-Methylcytosine may be further oxidized to 5-hydroxymethylcytosine. These two modifications are examples of distinct epigenetic marks, which can then be specifically recognized (“read-out”) with specific reader domains in many proteins.

Methyl-CpG binding domain (MBD) proteins were first identified in mammals that have very high CpG methylation levels as high as 70–80%^3^ as chromatin regulators that recognize the cytosine methylation at CpG dinucleotides (hereafter: methyl-CpG sites).^4,5^ Within the MBD family, several “canonical” MBD proteins from humans (*Homo sapiens*, Hs) have been intensively studied. For example, HsMeCP2 and HsMBD1 act as transcriptional repressors by binding to the methyl-CpG site and then recruiting histone methyltransferases and histone deacetylases, which promote chromatin condensation (i.e., formation of mature heterochromatin).^6^ As another example of a canonical MBD protein, the HsMBD4 MBD domain preferably recognizes (mismatch) TpG/methyl-CpG and hydroxymethyl-CpG/methyl-CpG sites, which are intermediate products in the course of active DNA demethylation.^7,8^ In addition to the MBD domain, these MBD proteins contain one or more functional domains that define the specific function of these proteins such as the transcription repression domain in HsMBD1 and the glycosylase domain in HsMBD4.^6^

In plants, cytosine bases in not only CpG but also symmetrical CpHpG and asymmetrical CpHpH contexts (H represents A, T, or C) can be methylated.^9,10^ All these methylation patterns are thought to be established by the RNA-directed DNA methylation mechanism and maintained through different regulatory pathways.^11,12^ Previous studies demonstrated that genome-wide levels of cytosine methylation in plants largely deviate among species; 24% CpG, 6.7% CpHpG, and 1.7% CpHpH are methylated in thale cress (*Arabidopsis thaliana*, At),^10^ whereas high methylation rates of 87–88% CpG, 67–68% CpHpG, and 41–43% CpHpH are observed in rice cultivars.^13^ Epigenetic mark reader domains, such as MBD proteins, are also found in plants. Interestingly, however, domain organization of plant MBD proteins is different from that of mammalian MBD proteins.^14^ The differences in the sequence contexts and the recognitive machineries of cytosine methylation between mammals and plants indicate that epigenetic regulation via DNA methylation in plant cells contains multiple pathways that do not exist in mammalian cells. However, the molecular mechanism by which each methylation pattern is read out, interpreted, and translated into downstream signals is still largely unclear.

In *A. thaliana*, 13 proteins, AtMBD1–AtMBD12 and AtIDM1, were identified as putative MBD proteins on the basis of their sequence homology with mammalian MBD proteins. Previous studies have shown by fluorescence microscopy that at least three of them, AtMBD5, AtMBD6, and AtMBD7, are localized to chromocenters abundant in cytosine methylation.^15^ Although the subnuclear distribution indicated that these MBD proteins recognize methylated DNA, both their binding specificity and affinity toward methylated DNA in various sequence contexts remain elusive. Furthermore, no structural information on the MBD domains from plants has been published.

Here, we focused on one particular MBD protein from *A. thaliana*, AtMBD6. AtMBD6 does not contain any known functional domains except the MBD domain. On the basis of the subnuclear colocalization observed by fluorescence microscopy and *in vitro* binding examined by pull-down assays, it has been speculated that AtMBD6 is involved in maintenance of DNA methylation mediated by an ATP-dependent DNA helicase, AtDDM1.^15^ Another previous study suggested by yeast two-hybrid assays and Förster resonance energy transfer experiments that AtMBD6 interacts with proteins involved in the RNA-directed DNA methylation pathway, including an RNA binding protein, AtAGO4, and a histone deacetylase AtHDA6.^16^ However, many fundamental properties of AtMBD6 such as the structure and the binding specificity toward methylated DNA remain to be elucidated. In the present study, we characterized the AtMBD6 MBD domain using NMR spectroscopy to establish a structural basis toward understanding the roles of MBD domains in the complex of epigenomic regulation in plant cells.

### Binding Specificity of the AtMBD6 MBD Domain toward Methylated DNA

**Figure 1:**
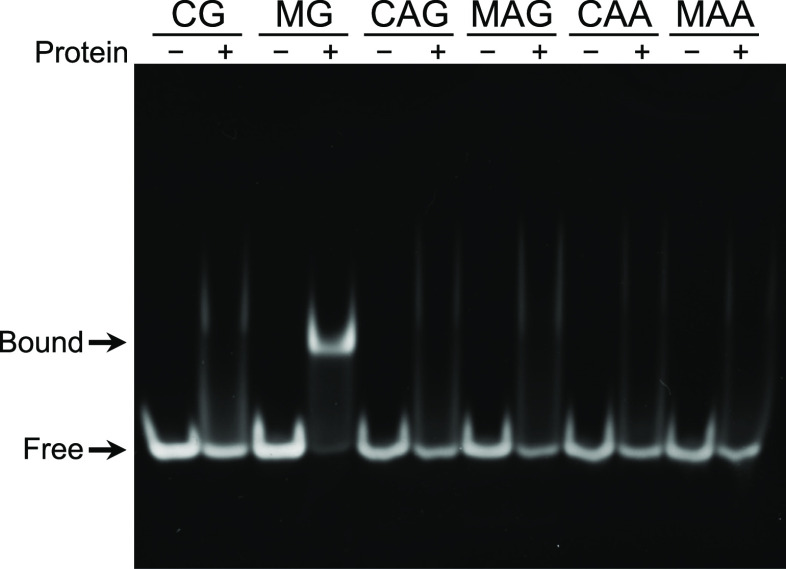
Binding specificity of the AtMBD6 MBD domain toward methylated DNA. Gel shift assay of DNA binding by MBD_AtMBD6_. The names of the DNA samples, indicated at the top of the panel, correspond to the sequences shown in Table 1.

First, we examined the binding preference of the MBD domain of AtMBD6 (residues 78–140; MBD_AtMBD6_) toward DNA harboring single CpG, CpApG, or CpApA sites in methylated or nonmethylated states (Table 1) using a gel shift assay. MBD_AtMBD6_ showed specific binding to the DNA harboring the methyl-CpG site and roughly the same degree of nonspecific binding to all other DNA samples examined (Figures 1 and S1). Since a previous study had indicated that AtMBD6 could form both a homodimer and a heterodimer with AtMBD5 *in vitro*,^17^ we considered that formation of a homodimer might alter the observed binding specificity of MBD_AtMBD6_ toward methylated DNA. However, the two-fold serial dilutions of a highly concentrated solution of MBD_AtMBD6_ showed no detectable changes in the backbone amide chemical shifts, indicating that MBD_AtMBD6_ exists as a monomer in solution even at concentrations as high as 200 μM (Figure S2). Taken together, these results indicated that MBD_AtMBD6_ is a canonical monomeric MBD domain that specifically recognizes the methyl-CpG sites in DNA.

**Table 1:** Sequences of the DNA Constructs Used in This Study

name	sequence^a^		
CG	5′	GGTATG**CG**CATACC	3′
	3′	CCATACGCGTATGG	5′
MG	5′	GGTATG**MG**CATACC	3′
	3′	CCATACGMGTATGG	5′
CAG	5′	GGTGAG**CAG**GATGC	3′
	3′	CCACTCGTCCTACG	5′
MAG	5′	GGTGAG**MAG**GATGC	3′
	3′	CCTCTCGTMCTACG	5′
CAA	5′	GGTGAG**CAA**GAGGC	3′
	3′	CCACTCGTTCTCCG	5′
MAA	5′	GGTGAG**MAA**GAGGC	3′
	3′	CCACTCGTTCTCCG	5′

a M represents 5-methylcytosine.

### Solution Structure of the AtMBD6 MBD Domain

**Figure 2:**
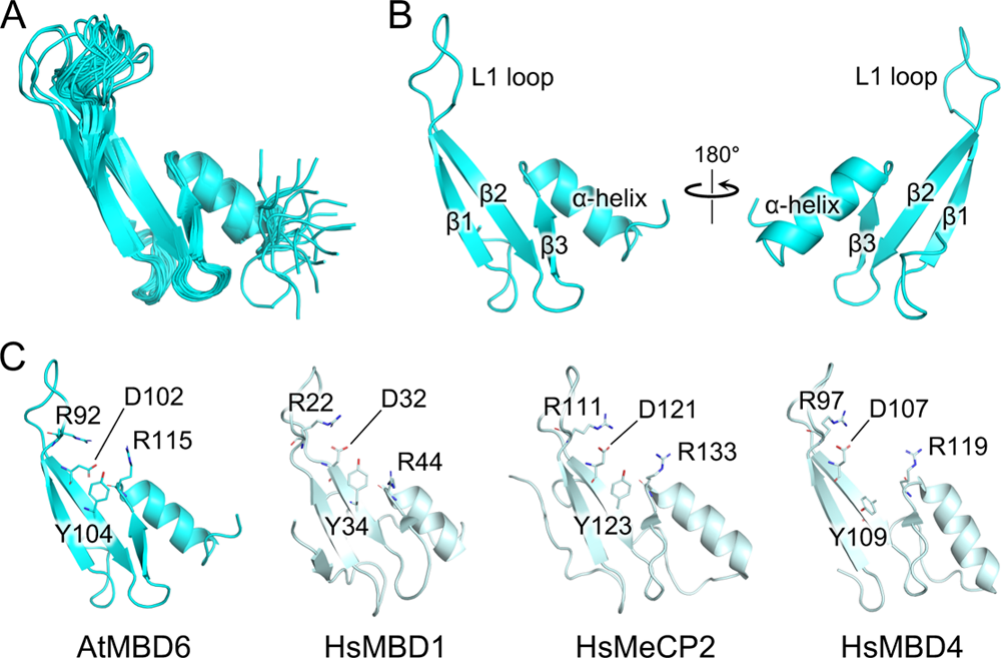
Solution structure of the AtMBD6 MBD domain. (A) Ensemble of the 20 minimum-energy structures of MBD_AtMBD6_. The plasmid-derived N-terminal peptide GPLGS and the disordered C-terminal residues 132–140 are omitted for clarity. (B) Front and back views of the lowest energy structure of MBD_AtMBD6_. (C) Comparison of the solution structure of MBD_AtMBD6_ in the free form with the crystal and solution structures of HsMBD1 (residues 4–69), HsMeCP2 (residues 91–162), and HsMBD4 (residues 86–140) in complex with methyl-CpG-containing DNA (Protein Data Bank IDs: 1IG4, 3C2I, and 2MOE). The DNA moieties are omitted for clarity. Four conserved residues that have been reported to be essential for specific recognition of the methyl-CpG sites in mammalian MBD domains are displayed as sticks. In the reported crystal structure of the HsMeCP2/methyl-CpG-containing DNA complex, these four residues play essential roles in methyl-CpG recognition as follows:^19^ R111 (RF1) and R133 (RF2) form hydrogen bonds with guanine bases of the methyl-CpG site. R133 also engages in hydrophobic interactions with the methyl group of one of the two 5-methylcytosines. The carbonyl group of D121 forms a weak hydrogen bond with the methyl group of the second 5-methylcytosine and is a part of a water-mediated hydrogen bond network, in which the methyl groups of both 5-methylcytosines are involved. D121 also stabilizes the RF1 by formation of a salt bridge. Y123 contributes to recognition of the two 5-methylcytosines by participating in the hydrogen bond network. In HsMBD4, which preferentially recognizes the TpG/methyl-CpG mismatch sites, the side chain of this tyrosine (Y109) is flipped out.

To gain structural insights into the DNA binding of MBD_AtMBD6_, we determined the solution structure of MBD_AtMBD6_ in the free form by NMR spectroscopy. In the initial structure calculation, interproton distance restraints from three-dimensional NOESY spectra and dihedral angle restraints derived from backbone and β-carbon chemical shifts were used. The lowest-energy structure of the NOE-based structure calculation was then used as the initial structure for further structural refinement applying residual dipolar coupling (RDC) restraints in addition. The obtained 20 minimum energy structures were well defined, as indicated by the root-mean-square deviation (RMSD) for the backbone of 0.6 ± 0.2 Å except for a loop region (residues 93–98) and a disordered C-terminal region (residues 128–140) (Figure 2A and Table 2).

**Table 2:** Statistics of the AtMBD6 MBD Domain NMR Structure Determination

distance restraints	
short range (|*i* – *j* | ≤ 1)	445
medium range (1 < |*i* – *j* | < 5)	125
long range (|*i* – *j* | ≥ 5)	164
total	734
^1^H–^15^N RDC restraints	37
RMSD statistics (residues 78–92, 99–127)	
backbone	0.6 ± 0.2 Å
heavy atoms	1.1 ± 0.1 Å
Ramachandran plot statistics (residues 78–127)	
residues in the most favored regions	89.2%
residues in additionally allowed regions	9.0%
residues in generously allowed regions	1.8%
residues in disallowed regions	0.0%

The structure is composed of three β-strands, an α-helix, and a flexible loop L1 that connects the β-strands β1 and β2 (Figure 2B). This fold is indeed the canonical fold of mammalian MBD domains. Accordingly, the overall structure is quite similar to the solution and crystal structures of mammalian MBD domains in complex with methyl-CpG-containing DNA reported previously (Figure 2C).^18−20^ The structural flexibility of the L1 loop is consistent with relatively small backbone heteronuclear NOE values previously observed for residues in this loop,^21^ and very similar observations have also been reported for the free forms of the MBD domains of HsMBD1 and HsMeCP2.^22,23^ In addition, four important residues including two arginine residues termed as arginine fingers (RFs), which are conserved among mammalian MBD domains, are also present at the corresponding position in the structure of MBD_AtMBD6_ (Figure 2C, shown as sticks). In the reported complexes of human MBD domains and DNA, these residues were shown to form unique structural motifs to recognize the methyl-CpG sites via hydrophobic interactions with the methyl groups of 5-methylcytosine bases and direct or water-mediated hydrogen bonds with 5-methylcytosine and guanine bases.^18,19,24^ Taken together, our data showed that MBD_AtMBD6_ adopts the canonical MBD fold in solution, which is in fine agreement with the result of the gel shift assay showing the binding specificity of MBD_AtMBD6_ toward methyl-CpG-containing DNA.

### Binding Interface and Affinity of the AtMBD6 MBD Domain for Methyl-CpG-Containing DNA

**Figure 3:**
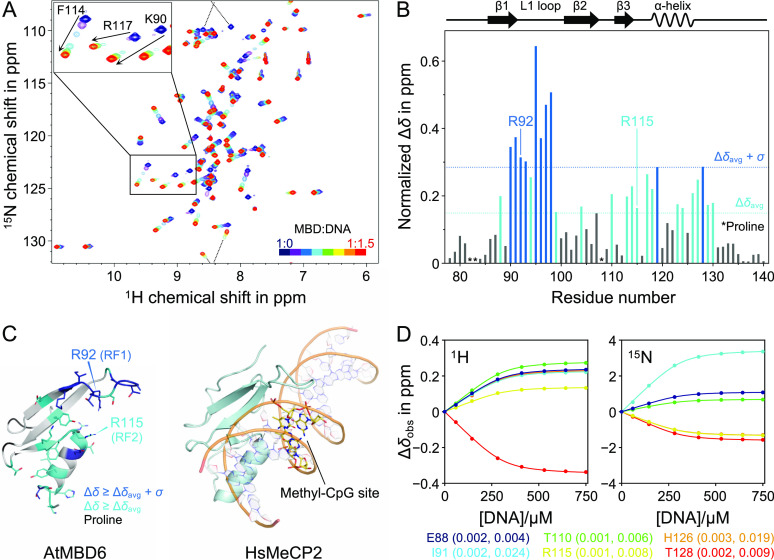
Binding interface and affinity of the AtMBD6 MBD domain for methyl-CpG-containing DNA. (A) Backbone amide chemical shift changes of MBD_AtMBD6_ during the course of titration. The dashed line indicates chemical shift changes across the spectral border (i.e., aliasing). As representative examples, three resonances that exhibited significant line broadening are shown in the enlarged inset. (B) Normalized CSD of each residue between the free state and the DNA-bound state. Δδ_avg_ and σ denote the averaged CSD value and the standard deviation, respectively. Schematic representation of the secondary structure is shown at the top. (C) Comparison of the binding interface of MBD_AtMBD6_ to the methyl-CpG site in DNA (left) with the crystal structure of the MBD domain of HsMeCP2 in complex with methyl-CpG-containing DNA (right). (D) Representative fitting curves of the chemical shift perturbation values of MBD_AtMBD6_ over the molar ratio during the course of titration. Fitting errors are shown as capped bars, although most of them are too small to see. The error values in Δδ_obs_ of ^1^H (left) and ^15^N (right) are also indicated in the parentheses of the residue labels. While only six examples are displayed in the panels, all backbone amide resonances were used in the fitting procedure to derive the global *K*_d_ value.

In order to reveal the mechanism of methyl-CpG recognition by MBD_AtMBD6_, we performed NMR titration experiments using ^15^N-labeled MBD_AtMBD6_ and unlabeled methyl-CpG-containing DNA as a ligand. Almost all cross-peaks of MBD_AtMBD6_ showed some degree of displacement upon the addition of DNA solution (Figure 3A). To examine whether any structural changes of MBD_AtMBD6_ occurred upon binding to methyl-CpG-containing DNA, we compared the secondary structure propensity (SSP) score of each residue in the free state^21^ and the bound state. No significant difference between the SSP scores of both forms was observed (Figure S3), suggesting that there are no major changes in the secondary structure of MBD_AtMBD6_ upon methyl-CpG-containing DNA binding. Next, we calculated the normalized chemical shift difference (CSD) values of all backbone amide resonances to understand which residues contribute most significantly to methyl-CpG-containing DNA binding. The binding surfaces were similar to those observed in the case of human MBD proteins (Figure 3B);^22,23^ the residues that displayed large CSD values are mainly located in the two RFs, the L1 loop, and the α-helix of which the corresponding regions in mammalian MBD proteins are in contact with methyl-CpG-containing DNA (Figure 3C).^8,19^ Notably, two residues in the L1 loop, G95 and A98, known as unique reporters for the methyl-CpG-specific binding mode of previously studied MBD domains,^25−27^ showed chemical shift changes very similar to the corresponding residues in these MBD domains (in terms of both directions and magnitude) upon interactions with methyl-CpG-containing DNA. All in all, our results indicate that MBD_AtMBD6_ uses a similar interface as conserved mammalian MBD domains to bind methyl-CpG-containing DNA.

Interestingly, several peaks exhibited significant line broadening when the molar ratio of DNA to MBD was in the range of 0.1–0.5 (Figure 3A, enlarged view). In addition, the magnitude of line broadening appeared to be roughly proportional to the degree of change in chemical shift upon binding. This observation indicated that MBD_AtMBD6_ binds to methyl-CpG-containing DNA in the fast to intermediate exchange regime on the NMR timescale; in general, this regime is associated with dissociation constants *K*_d_ ranging from several micromolar to several tens of micromolar.^28,29^ In order to determine the *K*_d_ value for this interaction, we fitted the chemical shift changes during the course of the titration to a two-state binding model (Figure 3D). The *K*_d_ value was obtained as 40.2 ± 0.5 μM, which is reasonable for the observed fast to intermediate exchange regime. Previous studies have revealed that several mammalian MBD domains show high affinity toward methyl-CpG-containing DNA with *K*_d_ values from several tens of nanomolar to a few micromolar.^24,30^ Therefore, our results clearly show that the binding affinity of MBD_AtMBD6_ toward methyl-CpG-containing DNA is much lower in comparison to that of the canonical MBD domains, even though MBD_AtMBD6_ shares the DNA binding interface with its mammalian counterparts.

### Consequence of Variation of Important Amino Acids in the AtMBD6 MBD Domain

**Figure 4:**
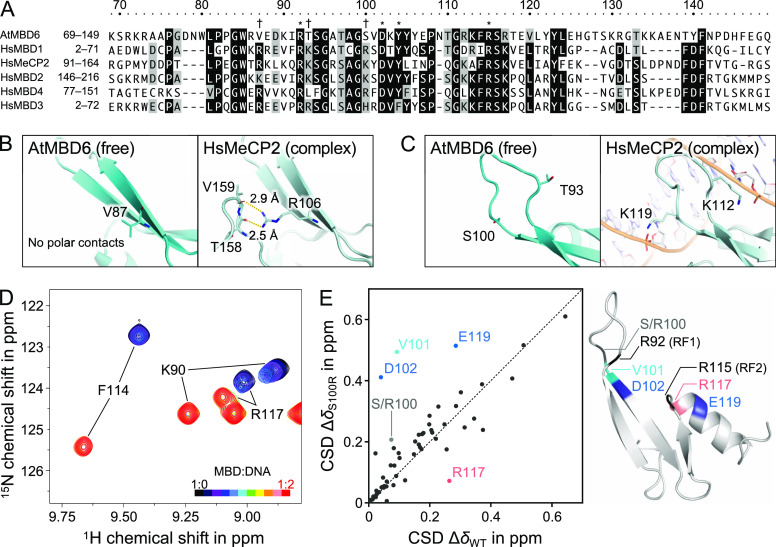
Consequence of variation of important amino acids in the AtMBD6 MBD domain. (A) Comparison of the amino acid sequence of MBD_AtMBD6_ with the sequences of human MBD domains. Identical and homologous residues are colored in black and gray, respectively. Asterisks indicate the key residues responsible for the specific binding of the canonical MBD domains to methyl-CpG-containing DNA. Daggers refer to the positively charged residues conserved among human MBD proteins but absent in AtMBD6. (B) Comparison of MBD_AtMBD6_ and the MBD domain of HsMeCP2 on the basis of presence and stabilization of the hairpin loop. Polar contacts are indicated by yellow dashed lines. In the description of the structure of HsMeCP2, the DNA moiety is omitted for clarity. (C) Comparison of MBD_AtMBD6_ and the MBD domain of HsMeCP2 on the coordinates of two important residues at the L1 loop that contact the DNA backbone. (D) Spectral changes of S100R MBD_AtMBD6_ upon binding to methyl-CpG-containing DNA (MG). For comparison to WT MBD_AtMBD6_, see also the enlarged inset in Figure 3A. (E) Correlation plot of the normalized CSD values upon binding between WT MBD_AtMBD6_ and the S100R mutant (left). Mapping of the residues displaying significant differences in the correlation on the structure of WT MBD_AtMBD6_ (right).

To understand the comparably weak binding of MBD_AtMBD6_ to methyl-CpG-containing DNA, we performed sequence and structural alignment of MBD_AtMBD6_ with human and other *A. thaliana* MBD domains (Figures 4A, S4, and S5). MBD_AtMBD6_ possesses all the key residues that have been shown to be responsible for specific recognition of the methyl-CpG sites in canonical MBD domains,^8,19^ in line with the aforementioned results. However, two critical differences were found in the amino acid sequences. First, the C-terminal region of MBD_AtMBD6_ (residues 127–149) shows poor homology with its human counterparts. Previous studies on human MBD domains demonstrated that this region forms a “hairpin loop” structure that is also associated with DNA binding.^23^ Therefore, we suppose that the affinity of MBD_AtMBD6_ toward DNA is reduced as a consequence of the absence of the hairpin loop. Second, three positively charged residues conserved among human MBD proteins (R17, K23, and R30 in HsMBD1) correspond to uncharged residues (V87, T93, and S100, respectively) in AtMBD6. In canonical human MBD proteins, the first of these three residues stabilizes the hairpin loop by electrostatic interactions with backbone carbonyl groups in the hairpin loop (Figure 4B).^19^ The absence of this positively charged residue therefore supports our idea that the hairpin loop is missing in MBD_AtMBD6_, that is, not only in the free but also even in the DNA-bound state. The remaining two residues are located near the two ends of the L1 loop and contact the phosphate backbone of DNA in the reported structures of canonical MBD-DNA complexes (Figure 4C).^18,19^ Previous studies demonstrated that the positive charge of these residues plays a crucial role in the DNA binding by human and chicken MBD domains.^18,23,31^ Therefore, we hypothesized that the absence of these important electrostatic contributions to the binding energy significantly reduces the binding affinity of MBD_AtMBD6_ toward methyl-CpG-containing DNA.

To explore this hypothesis, we constructed a single-point mutant S100R MBD_AtMBD6_ and conducted NMR titration experiments under the same conditions as performed with wildtype (WT) MBD_AtMBD6_. As the molar ratio of DNA to MBD was raised, cross-peaks of S100R MBD_AtMBD6_ corresponding to the free state gradually disappeared with only very slight changes in the chemical shift, while the peak intensity of the bound state increased (Figures 4D and S6A). Several cross-peaks of the free state did not move straight toward the bound state, suggesting that an intermediate state detectable on the chemical shift timescale might be involved in the DNA binding of S100R MBD_AtMBD6_. These observations indicated that the off-rate of the binding was lower than that of WT MBD_AtMBD6_ and the exchange between the intermediate and bound states was in the slow exchange regime on the chemical shift timescale. Taken together, our results suggest that the S100R mutant has an increased affinity toward methyl-CpG-containing DNA as compared with WT MBD_AtMBD6_, in line with the hypothesis based on the sequence alignment. To obtain the *K*_d_ value of this interaction, we tried several methods including this NMR titration, isothermal titration calorimetry, and fluorescence polarization. Unfortunately, however, we could not obtain a reliable *K*_d_ value, likely because this interaction is not a simple two-state exchange and several properties of this interaction and MBD_AtMBD6_ itself such as binding enthalpy and molecular weight are not suitable for quantification of the binding affinity by these methods. Therefore, hereafter, we focus on the differences in the chemical shift change upon DNA binding between WT and S100R MBD_AtMBD6_. For most of the residues, the CSD values between the free and bound states were similar to the corresponding CSD values of WT MBD_AtMBD6_; however, the four residues (V101, D102, R117, and E119) showed large differences in the chemical shift change upon DNA binding between WT and S100R MBD_AtMBD6_ (Figure 4E, left panel; see also Figures 3B and S6B). Among these four residues, only R117 experienced a “downward” change, suggesting that this residue is less involved in the DNA binding in the S100R mutant compared to WT MBD_AtMBD6_. By contrast, V101, D102, and E119 showed “upward” changes, suggesting that these residues became more involved in DNA binding due to the introduction of the S100R mutation. It should also be noted that R117 and E119 are not close to the mutation site (more than 10 Å apart from S/R100). Intriguingly, we found that these four residues are located near either of the RFs (Figure 4E, right). Overall, the main distinction between the CSD values of WT and S100R MBD_AtMBD6_ results from the difference in the chemical shifts of the bound state, not the free state (Figures S6C,D). Furthermore, as the molar ratio of DNA over protein increased, an aliased side-chain signal emerged in the ^1^H–^15^N HSQC spectra at a markedly downfield-shifted ^1^H chemical shift (Figure S6A). Based on a ^15^N-edited NOESY-HSQC spectrum, this resonance could be identified as the ε-NH resonance of R92 of the structural element RF1. By contrast, this signal was not observed in the case of WT MBD_AtMBD6_, further underlining the differential involvement of RF1 in WT and S100R MBD_AtMBD6_. Thus, taken together, these results suggest that “exchanging” positively charged residues responsible for electrostatic interactions with the DNA backbone (in human MBD domains) to uncharged residues (in MBD_AtMBD6_) specifically affects local conformational states around the two distinct RFs in the bound state. In other words, the reduced binding affinity of MBD_AtMBD6_ toward methyl-CpG-containing DNA can be considered a result of the lack of important nonspecific interactions and the consequent alterations in the DNA binding mode of the RFs that are responsible for the binding specificity.

## Discussion

### Structural Properties of the AtMBD6 MBD Domain

Among the 13 MBD proteins from *A. thaliana*, AtMBD6, AtMBD5, and AtMBD7 show the highest sequence homology in the MBD domain with mammalian MBD domains that specifically recognize the methyl-CpG sites.^32^ Correspondingly, the overall structure of MBD_AtMBD6_ in the free form is almost identical to the canonical structures of known mammalian MBD domains, except for the C-terminal region. The insertion of a single residue, N109, in the short loop between β2 and β3 of MBD_AtMBD6_ (Figure 4A) has almost no effect on the overall structure of the MBD core (Figure 2). On the basis of sequence homology, other MBD proteins from *A. thaliana* also seem to adopt the canonical MBD fold, although one or more residues in the L1 loop are missing except for AtMBD5 and AtMBD8 that do possess these residues (i.e., they have a “full” L1 loop).^33,34^

While we found that the L1 loop of MBD_AtMBD6_ shows high structural flexibility prior to DNA binding, interestingly, previous structural and chemical shift analyses indicated that the specific binding of human MBD domains to the methyl-CpG site stabilizes the dynamic L1 loop, thereby reducing its conformational flexibility.^18,25^ Since the amino acid composition in this region of AtMBD6 is similar to that of human MBD proteins, the L1 loop of MBD_AtMBD6_ would also recognize the major groove of DNA by hydrophobic interactions and hydrogen bonds to become rigid upon binding to methyl-CpG-containing DNA.^18^

The C-terminal region of MBD_AtMBD6_ shows no noticeable sequence homology with human MBD domains and was found to be highly disordered in the solution structure, in fine agreement with the negative backbone heteronuclear NOE values reported in our initial study on MBD_AtMBD6_.^21^ This implies that the requirements for formation of a stabilized hairpin loop in plant MBD domains are the same as in human MBD domains: both a tripeptide motif FBF (B represents D or N) at the respective position in the C-terminal region (see Figure 4A) and a positively charged residue that forms polar contacts with the hairpin loop at the beginning of β1. According to amino acid sequence similarity (Figure S4), MBD proteins from *A. thaliana* can be classified into two groups by the presence and absence of the hairpin loop. However, our results indicate that the presence or absence of the hairpin loop alone does not correlate with the binding ability of the MBD domains to methyl-CpG-containing DNA in *A. thaliana*.

### DNA Binding Specificity of the AtMBD6 MBD Domain

The hitherto published reports of DNA binding specificity of AtMBD6, which were determined on the basis of gel shift assay experiments, are not in mutual agreement, for example, one group claims that AtMBD6 binds all types of methylated DNA in plants,^35^ whereas other groups claim that AtMBD6 specifically recognizes the methyl-CpG sites.^32,34^ Possible reasons for this discrepancy include artifacts derived from the unstructured regions of AtMBD6, the presence of an N-terminal affinity tag, and differences in the experimental buffer composition. To resolve this contradiction, we had prepared high-purity tag-free MBD_AtMBD6_ samples and qualitatively evaluated its DNA binding specificity using all types of methylated DNA at the same time, that is, on the same gel. Under the conditions employed here, MBD_AtMBD6_ specifically recognized methyl-CpG-containing DNA, suggesting that AtMBD6 is not directly involved in epigenetic regulation via methylation in CpHpG and CpHpH contexts. With respect to methyl-CpHpG and methyl-CpHpH contexts, previous reports had also revealed that these sequences are recognized by plant-specific histone methyltransferase SUVH family proteins that contain the SET- and RING-associated domains.^36−38^ Thus, we speculate that the MBD and SUVH family proteins might function in different signaling pathways distinguished by DNA methylation patterns in plant cells.

### Mechanism of Weak Binding of AtMBD6 to Methyl-CpG-Containing DNA

**Figure 5:**
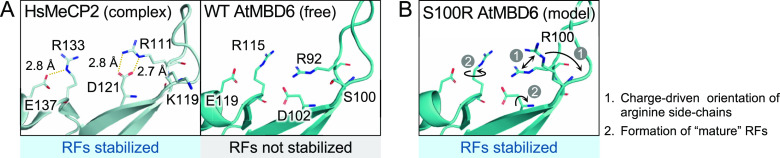
Mechanism of weak binding of AtMBD6 to methyl-CpG-containing DNA. (A) Comparison of WT MBD_AtMBD6_ and the MBD domain of HsMeCP2 showing, respectively, the presence and absence of salt bridges stabilizing the two RFs. Polar contacts are indicated by yellow dashed lines. In the description of the structure of HsMeCP2, the DNA moiety is omitted for clarity. Note that the orientations of the side chains of the two RFs are not completely defined in WT MBD_AtMBD6_ in the free state; however, our results indicate that the orientations of the RFs are still not entirely fixed in the DNA-bound state. (B) Model of the conformational changes upon DNA binding in S100R MBD_AtMBD6_. Repulsion of positive charges between R92 (RF1) and R100 retains the conformation of R92 and directs R100 toward the DNA backbone (shown as “1”). This coordinated effect enables formation of a stable salt bridge between R92 and D102, which cooperatively induces maturation of the RF2 motif by the electrostatic stabilization between R115 and E119 (shown as “2”).

We revealed by solution NMR spectroscopy that MBD_AtMBD6_ possesses a similar binding interface to methyl-CpG-containing DNA as canonical mammalian MBD domains, albeit with one to three orders of magnitude lower binding affinity. One of the reasons for the comparably low affinity of MBD_AtMBD6_ is the absence of two positively charged residues that have been shown to electrostatically interact with the DNA backbone in other MBD domains (T93 and S100 in AtMBD6).^18,19^ NMR titration experiments using a single-point mutant to “correct” one of these atypical residues, S100R, indicated that the difference between MBD_AtMBD6_ and canonical MBD domains lies in the chemical environment in the vicinity of the two RFs (V101, D102, R117, and E119 in AtMBD6) in the DNA-bound state. However, the backbone amide CSD values of the RFs themselves were not significantly changed by the S100R mutation (see Figure S6D), indicating that only their side-chain guanidino groups might be involved in the mechanistic difference in DNA binding affinity between AtMBD6 and canonical MBD proteins. A simple effect of the S100R mutation is an attractive electrostatic interaction between the side chain of the introduced arginine R100 and the DNA backbone, as observed in the reported structures of canonical MBD domains in complex with methyl-CpG-containing DNA. Another possible aspect of this mutation may be an electrostatic repulsion between the positively charged guanidino groups of R100 and the RF1, that is, R92. These effects seem to explain the observed changes in the vicinity of RF1 but not in RF2. Importantly, D121 and E137 in HsMeCP2 corresponding to D102 and E119 in AtMBD6 form salt bridges with the guanidino groups of the two RFs to fix them in appropriate positions for specific recognition of the methyl-CpG site in the complex (Figure 5A, left panel).^19^ Therefore, the observed differences between WT MBD_AtMBD6_ and the S100R mutant indicate that the absence of important local interactions with the DNA backbone in MBD_AtMBD6_ makes the retention of RF1 and RF2 (cooperative binding) in a proper conformation insufficient (Figure 5A, right panel, Figure 5B). Indeed, the downfield-shifted RF1 side-chain resonance is reminiscent of the methyl-CpG-specific binding mode exhibited by the RF1 motif of several MBD domains from human and even the invertebrate *Ephydatia muelleri*.^25,26^ The facts that in the complex, the side chain of D121 in HsMeCP2 also forms a polar contact with its backbone amide group (3.2 Å distance between one of the two carboxy oxygen atoms and the amide nitrogen atom) and that D102 in WT MBD_AtMBD6_ showed only a small CSD value upon DNA binding support our conclusion that the RF1 motif is not properly formed in WT MBD_AtMBD6_. Collectively, the conformational incompleteness of the RF motifs in AtMBD6 in the complex appears to increase the off-rate, resulting in a significant reduction in the binding affinity toward methyl-CpG-containing DNA, although we stress again that MBD_AtMBD6_ does still preserve the binding *specificity*.

Our analysis further indicates that the DNA binding affinity of AtMBD5 is likely to be even lower than that of AtMBD6, as the important tyrosine (Y104 in AtMBD6) is substituted to phenylalanine (Figures 2C and S4) and, thus, AtMBD5 could not form the water-mediated hydrogen bond network that has been implicated in DNA base recognition by HsMeCP2.^19^ The sequence homology of the MBD domains of AtMBD7 with mammalian MBD domains is slightly lower than that of AtMBD6. Therefore, the DNA binding affinity of each individual MBD unit is likely to be even lower than that of AtMBD6. However, AtMBD7 may still retain a moderate binding affinity toward methyl-CpG-containing DNA by cooperative binding of the three tandem MBD domains to multiple methyl-CpG sites.

### Possible Mechanism of Subnuclear Localization of AtMBD6 Harboring an MBD Domain with Low DNA Binding Affinity

Although the binding affinity of the MBD domain toward methyl-CpG-containing DNA is significantly reduced, AtMBD6 localizes to highly methylated heterochromatin.^15,34^ Given that 24% of the CpG sites in genomic DNA are methylated in *A. thaliana*,^10^ the density of the methyl-CpG sites may aid proper subnuclear localization of AtMBD6 even with a comparably low DNA binding affinity. In addition, regions other than the MBD domain itself might also contribute to binding of AtMBD6 toward methyl-CpG-containing DNA, although at present, no significant sequence homology with any known DNA-binding domain is detected for the N- or C-terminal regions of AtMBD6.

Another possible explanation for the localization of AtMBD6 to heterochromatin would be that binding partners compensate for the low DNA binding affinity of AtMBD6. One of the putative AtMBD6-binding proteins, AtDDM1, possesses a DNA helicase domain and was shown to bind to both free DNA and nucleosomal DNA.^39^ Another potential binding partner of AtMBD6, AtAGO4, plays a crucial role in the RNA-directed DNA methylation^9^ and thus might also bind to DNA with sequence preference. Indeed, a previous study showed by fluorescence recovery analysis that AtMBD6 is highly mobile at perinucleolar chromocenters, while a small fraction of the protein is relatively immobile.^17^ We assume that the immobile fraction that tightly binds to chromatin is involved in formation of protein–protein complexes and the mobile fraction corresponds to the free molecules.

## Conclusions

In this study, we revealed that MBD_AtMBD6_ specifically recognizes methyl-CpG sites and characterized its structural properties at the atomic level. MBD_AtMBD6_ binds methyl-CpG-containing DNA with a significantly reduced affinity compared to mammalian MBD domains, while its DNA binding interface is conserved. Future structural studies of AtMBD6 in complex with methyl-CpG-containing DNA and analysis of its interactions with previously identified binding partners ought to pave the way of understanding the mechanism by which AtMBD6 serves as a repressor of gene expression in plant cells.

## Materials and Methods

### Sample Preparation

The expression vector pGEX-6P-1 encoding the MBD domain of AtMBD6 (UniProtKB entry Q9LTJ1, residues 78–140) was used for protein overexpression in bacterial cells. Site-directed mutagenesis to generate S100R MBD_AtMBD6_ was achieved by inverse PCR. Proteins were prepared as previously described^21^ with a few modifications. Briefly, *Escherichia coli* BL21(DE3) cells carrying the expression plasmids were grown in Lennox’s LB media or M9 minimal media containing 2 g/L uniformly ^13^C-labeled d-glucose (Cambridge Isotope Laboratories) or 4 g/L unlabeled d-glucose (Nacalai Tesque), 1 g/L ^15^N-labeled ammonium chloride (Cambridge Isotope Laboratories), and as antibiotic selection pressure 100 mg/L ampicillin. Proteins were expressed for 20 h at 16 °C and purified using glutathione affinity chromatography. After on-column digestion of the N-terminal glutathione *S*-transferase-tag by HRV3C protease overnight at 4 °C, proteins were eluted from the column and further purified by size-exclusion chromatography. Purity and integrity of the samples were verified by SDS-PAGE and MALDI-TOF mass spectrometry. The yields of proteins per 1 L culture were 4–5 mg for LB and 2–4 mg for M9.

All DNA samples used in this study were purchased from Hokkaido System Science. These DNA sequences are summarized in Table 1. Single-stranded DNA samples were dissolved in 10 mM Tris buffer (pH 8.0 at 4 °C) containing 50 mM sodium chloride for the gel shift assay or in the titration buffer containing 20 mM sodium phosphate (pH 6.5 at 25 °C) and 150 mM sodium chloride for NMR titration experiments. Annealing was performed by incubating the solutions for 3 min at 98 °C and then gradually lowering the temperature to 4 °C over a total duration of 140 min.

### DNA Binding Assay

The mixture of 80 pmol annealed DNA (final 4 μM) and 400 pmol protein (final 20 μM) was incubated in 20 μL of the binding buffer containing 25 mM Tris, 25 mM boric acid, 150 mM sodium chloride, 5% glycerol, and 1 mM dithiothreitol for 30 min at 4 °C. Then, 5 μL of the reaction sample was loaded onto an 8% polyacrylamide gel containing 25 mM Tris and 25 mM boric acid and subjected to electrophoresis at 150 V for 60 min at 4 °C (i.e., in the cold room). Double-stranded DNA was stained with ethidium bromide, and the corresponding fluorescent bands were detected by UV irradiation using a ChemiDoc XRS Plus system (Bio-Rad).

### General NMR Spectroscopy

All NMR experiments were performed at 20 °C using an AVANCE II 700 MHz NMR spectrometer (Bruker) equipped with a TCI cryogenic probe with the exception of in-phase/anti-phase (IPAP) HSQC experiments, which were performed on an AVANCE 600 MHz NMR spectrometer (Bruker) equipped with a TXI cryogenic probe. NMR samples contained 5% deuterium oxide (Cambridge Isotope Laboratories) and were measured in 5 mm-diameter Shigemi tubes (Shigemi). Sodium 3-(trimethylsilyl)-1-propanesulfonate (Tokyo Chemical Industry) was used as an external standard of the ^1^H chemical shift; ^13^C and ^15^N chemical shifts were calibrated indirectly.^40^ All acquired NMR data (i.e., free induction decays) were processed using NMRPipe,^41^ and the resulting spectra were further analyzed using various software packages (see below).

### NMR Analysis of the Homo-oligomerization Status of the AtMBD6 MBD Domain

Two-dimensional ^1^H–^15^N HSQC spectra of ^13^C, ^15^N-labeled MBD_AtMBD6_ in the titration buffer were acquired with sequentially decreasing the concentration of the protein by twofold dilution. The concentration of MBD_AtMBD6_ in these experiments ranged from 200 to 3.1 μM. Analysis of the obtained spectra was performed using CcpNmr Analysis.^42^

### NOESY Experiments

Three-dimensional ^15^N-edited NOESY-HSQC (8417.509 Hz/512, 1773.679 Hz/24, and 8417.509 Hz/64), ^13^C-edited aliphatic NOESY-HSQC (8417.509 Hz/512, 7394.722 Hz/32, and 8417.509 Hz/64), and ^13^C-edited aromatic NOESY-HSQC (8417.509 Hz/512, 4225.978 Hz/24, and 8417.509 Hz/64) spectra were obtained using 1.5 mM ^13^C, ^15^N-labeled MBD_AtMBD6_ dissolved in the NMR buffer containing 20 mM sodium phosphate (pH 6.5 at 25 °C) and 50 mM sodium chloride; the numbers in the parentheses indicate, respectively, the spectral widths and the number of complex points in the *F*_3_, *F*_2_, and *F*_1_ dimensions. Each experiment was performed with a mixing time of 150 ms. NOE peaks were picked automatically, and the peak lists were refined manually using MagRO^43^ on NMRView.^44^

### IPAP HSQC Experiments

IPAP ^1^H–^15^N HSQC experiments were performed using 0.5 mM ^13^C, ^15^N-labeled MBD_AtMBD6_ samples in the presence and absence of pentaethylene glycol monododecyl ether (C_12_E_5_) bicelles as alignment medium.^45^ The bicelle solution was prepared by gradually adding a total of 15 μL 1-hexanol (Tokyo Chemical Industry) to a solution composed of 50 μL of C_12_E_5_ (Sigma-Aldrich), 200 μL of the NMR buffer, and 50 μL of deuterium oxide. The final concentration of C_12_E_5_ in the NMR sample was 4%. IPAP HSQC (*F*_2_: 7246.377 Hz/512 complex points, *F*_1_: 1520.447 Hz/350 complex points) experiments were conducted consecutively twice for each sample. Each acquired IPAP spectrum was split into two distinct spectra (IP + AP and IP – AP) in TopSpin (Bruker) and analyzed with CcpNmr Analysis. The averaged backbone amide RDC constants were used to generate RDC restraints in the structure refinement.

### Structure Determination of the AtMBD6 MBD Domain in the Free Form

The initial structure calculation of MBD_AtMBD6_ in the free form was performed using CYANA^46^ with interproton distance restraints derived from NOESY spectra (NOE peaks were automatically assigned based on the chemical shifts of backbone and side-chain resonances reported previously^21^) and backbone dihedral angle restraints estimated by TALOS+.^47^ The lowest-energy structure of the 20 structures calculated using CYANA was used as an initial structure in the subsequent refinement procedure. Structure refinement was performed using 500 cycles of a combination of simulated annealing and energy minimization using XPLOR-NIH^48,49^ while additionally applying RDC-based restraints. The top 20 structures of the 41 structures that meet the acceptance criteria are reported here with the statistics shown in Table 2. The coordinates of the solution structure of MBD_AtMBD6_ in the free form were deposited in the Protein Data Bank with accession ID 7D8K.

### NMR Titration and Resonance Assignment of the AtMBD6 MBD Domain in the Bound State

A solution of 4.5 mM double-stranded methyl-CpG-containing DNA termed as MG (see Table 1) was added in a stepwise manner to 0.6 mM ^13^C, ^15^N-labeled WT MBD_AtMBD6_ or ^15^N-labeled S100R MBD_AtMBD6_ in the titration buffer. After each addition of the ligand solution, a ^1^H–^15^N HSQC spectrum was acquired. To obtain backbone resonance assignments of WT MBD_AtMBD6_ in the bound state, HNCO, HN(CA)CO, CBCA(CO)NH, and HNCACB experiments^50^ were carried out after the final titration experiment. The triple resonance spectra were analyzed using MagRO-NMRView, and initial assignments were performed using FLYA,^51^ followed by manual verification and correction of the automated assignments. For each residue, the SSP score was evaluated from its chemical shifts of amide proton, amide nitrogen, α-carbon, β-carbon, and carbonyl carbon with the SSP program.^52^ In the case of the S100R mutant, resonances of backbone amide groups were assigned using three-dimensional ^15^N-edited NOESY-HSQC spectra with CcpNmr Analysis. The normalized CSD value was calculated as follows^53^
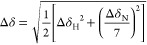
in which Δδ_H_ and Δδ_N_ are the CSD values in the ^1^H and ^15^N dimensions of the ^1^H–^15^N HSQC spectra, respectively. The titration curves of WT MBD_AtMBD6_ were fitted to a classical two-state binding model using GLOVE^54^ to derive the dissociation constant *K*_d_ as a global parameter. In this model, the observed chemical shift change Δδ_obs_ (where Δδ_obs_ is quantified separately for the ^1^H and ^15^N dimensions) is represented as follows^28^
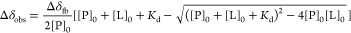
in which Δδ_fb_ is the CSD between the free state and bound state. [P]_0_ and [L]_0_ denote the total concentrations of the protein and ligand, respectively. Fitting errors were estimated by 100 steps of Monte Carlo simulation implemented in GLOVE.

## Supporting Information Available

Gel shift assay of DNA binding by the AtMBD6 MBD domain at different protein concentrations; overlaid ^1^H–^15^N HSQC spectrum of the AtMBD6 MBD domain at different concentrations; comparison of the SSP scores of the AtMBD6 MBD domain in the free and DNA-bound states; sequence alignment of the MBD domains in *A. thaliana*; structural superposition of the AtMBD6 MBD domain and human MBD domains; and details on the binding of the S100R AtMBD6 MBD domain to methyl-CpG-containing DNA (PDF)

## Accession Codes

The UniProtKB entry ID of MBD6 from *A. thaliana* is Q9LTJ1. The atomic coordinates of the solution structure of the AtMBD6 MBD domain in the free form were deposited in the Protein Data Bank as an entry 7D8K.

## References

[ref1] Goll M. G.; Bestor T. H. Eukaryotic Cytosine Methyltransferases. Annu. Rev. Biochem. 2005, 74, 481–514. 10.1146/annurev.biochem.74.010904.153721. 15952895

[ref2] Law J. A.; Jacobsen S. E. Establishing, Maintaining and Modifying DNA Methylation Patterns in Plants and Animals. Nat. Rev. Genet. 2010, 11, 204–220. 10.1038/nrg2719. 20142834 PMC3034103

[ref3] Li E.; Zhang Y. DNA Methylation in Mammals. Cold Spring Harbor Perspect. Biol. 2014, 6, a019133 10.1101/cshperspect.a019133. PMC3996472 24789823

[ref4] Meehan R. R.; Lewis J. D.; McKay S.; Kleiner E. L.; Bird A. P. Identification of a Mammalian Protein That Binds Specifically to DNA Containing Methylated CpGs. Cell 1989, 58, 499–507. 10.1016/0092-8674(89)90430-3. 2758464

[ref5] Hendrich B.; Bird A. Identification and Characterization of a Family of Mammalian Methyl-CpG Binding Proteins. Mol. Cell. Biol. 1998, 18, 6538–6547. 10.1128/mcb.18.11.6538. 9774669 PMC109239

[ref6] Du Q.; Luu P.-L.; Stirzaker C.; Clark S. J. Methyl-CpG-Binding Domain Proteins: Readers of the Epigenome. Epigenomics 2015, 7, 1051–1073. 10.2217/epi.15.39. 25927341

[ref7] Hendrich B.; Hardeland U.; Ng H.-H.; Jiricny J.; Bird A. The Thymine Glycosylase MBD4 Can Bind to the Product of Deamination at Methylated CpG Sites. Nature 1999, 401, 301–304. 10.1038/45843. 10499592

[ref8] Otani J.; Arita K.; Kato T.; Kinoshita M.; Kimura H.; Suetake I.; Tajima S.; Ariyoshi M.; Shirakawa M. Structural Basis of the Versatile DNA Recognition Ability of the Methyl-CpG Binding Domain of Methyl-CpG Binding Domain Protein 4. J. Biol. Chem. 2013, 288, 6351–6362. 10.1074/jbc.M112.431098. 23316048 PMC3585070

[ref9] Henderson I. R.; Jacobsen S. E. Epigenetic Inheritance in Plants. Nature 2007, 447, 418–424. 10.1038/nature05917. 17522675

[ref10] Cokus S. J.; Feng S.; Zhang X.; Chen Z.; Merriman B.; Haudenschild C. D.; Pradhan S.; Nelson S. F.; Pellegrini M.; Jacobsen S. E. Shotgun Bisulphite Sequencing of the Arabidopsis Genome Reveals DNA Methylation Patterning. Nature 2008, 452, 215–219. 10.1038/nature06745. 18278030 PMC2377394

[ref11] Matzke M. A.; Mosher R. A. RNA-Directed DNA Methylation: An Epigenetic Pathway of Increasing Complexity. Nat. Rev. Genet. 2014, 15, 394–408. 10.1038/nrg3683. 24805120

[ref12] Zhang H.; Lang Z.; Zhu J.-K. Dynamics and Function of DNA Methylation in Plants. Nat. Rev. Mol. Cell Biol. 2018, 19, 489–506. 10.1038/s41580-018-0016-z. 29784956

[ref13] Garg R.; Narayana Chevala V.; Shankar R.; Jain M. Divergent DNA Methylation Patterns Associated with Gene Expression in Rice Cultivars with Contrasting Drought and Salinity Stress Response. Sci. Rep. 2015, 5, 14922 10.1038/srep14922. 26449881 PMC4598828

[ref14] Springer N. M.; Kaeppler S. M. Evolutionary Divergence of Monocot and Dicot Methyl-CpG-Binding Domain Proteins. Plant Physiol. 2005, 138, 92–104. 10.1104/pp.105.060566. 15888682 PMC1104165

[ref15] Zemach A.; Li Y.; Wayburn B.; Ben-Meir H.; Kiss V.; Avivi Y.; Kalchenko V.; Jacobsen S. E.; Grafi G. DDM1 Binds Arabidopsis Methyl-CpG Binding Domain Proteins and Affects Their Subnuclear Localization. Plant Cell 2005, 17, 1549–1558. 10.1105/tpc.105.031567. 15805479 PMC1091773

[ref16] Parida A. P.; Sharma A.; Sharma A. K. AtMBD6, a Methyl CpG Binding Domain Protein, Maintains Gene Silencing in Arabidopsis by Interacting with RNA Binding Proteins. J. Biosci. 2017, 42, 57–68. 10.1007/s12038-016-9658-1. 28229965

[ref17] Zemach A.; Gaspan O.; Grafi G. The Three Methyl-CpG-Binding Domains of AtMBD7 Control Its Subnuclear Localization and Mobility. J. Biol. Chem. 2008, 283, 8406–8411. 10.1074/jbc.M706221200. 18211904 PMC2417168

[ref18] Ohki I.; Shimotake N.; Fujita N.; Jee J.-G.; Ikegami T.; Nakao M.; Shirakawa M. Solution Structure of the Methyl-CpG Binding Domain of Human MBD1 in Complex with Methylated DNA. Cell 2001, 105, 487–497. 10.1016/s0092-8674(01)00324-5. 11371345

[ref19] Ho K. L.; McNae I. W.; Schmiedeberg L.; Klose R. J.; Bird A. P.; Walkinshaw M. D. MeCP2 Binding to DNA Depends upon Hydration at Methyl-CpG. Mol. Cell 2008, 29, 525–531. 10.1016/j.molcel.2007.12.028. 18313390

[ref20] Walavalkar N. M.; Cramer J. M.; Buchwald W. A.; Scarsdale J. N.; Williams D. C. Solution Structure and Intramolecular Exchange of Methyl-Cytosine Binding Domain Protein 4 (MBD4) on DNA Suggests a Mechanism to Scan for MCpG/TpG Mismatches. Nucleic Acids Res. 2014, 42, 11218–11232. 10.1093/nar/gku782. 25183517 PMC4176167

[ref21] Iwakawa N.; Mahana Y.; Ono A.; Ohki I.; Walinda E.; Morimoto D.; Sugase K.; Shirakawa M. Backbone and Side-Chain Resonance Assignments of the Methyl-CpG-Binding Domain of MBD6 from Arabidopsis Thaliana. Biomol. NMR Assignments 2019, 13, 59–62. 10.1007/s12104-018-9851-2. 30242623

[ref22] Wakefield R. I. D.; Smith B. O.; Nan X.; Free A.; Soteriou A.; Uhrin D.; Bird A. P.; Barlow P. N. The Solution Structure of the Domain from MeCP2 That Binds to Methylated DNA. J. Mol. Biol. 1999, 291, 1055–1065. 10.1006/jmbi.1999.3023. 10518942

[ref23] Ohki I.; Shimotake N.; Fujita N.; Nakao M.; Shirakawa M. Solution Structure of the Methyl-CpG-Binding Domain of the Methylation-Dependent Transcriptional Repressor MBD1. EMBO J. 1999, 18, 6653–6661. 10.1093/emboj/18.23.6653. 10581239 PMC1171728

[ref24] Liu K.; Xu C.; Lei M.; Yang A.; Loppnau P.; Hughes T. R.; Min J. Structural Basis for the Ability of MBD Domains to Bind Methyl-CG and TG Sites in DNA. J. Biol. Chem. 2018, 293, 7344–7354. 10.1074/jbc.RA118.001785. 29567833 PMC5949999

[ref25] Cramer J. M.; Scarsdale J. N.; Walavalkar N. M.; Buchwald W. A.; Ginder G. D.; Williams D. C. Probing the Dynamic Distribution of Bound States for Methylcytosine-Binding Domains on DNA. J. Biol. Chem. 2014, 289, 1294–1302. 10.1074/jbc.M113.512236. 24307175 PMC3894315

[ref26] Cramer J. M.; Pohlmann D.; Gomez F.; Mark L.; Kornegay B.; Hall C.; Siraliev-Perez E.; Walavalkar N. M.; Sperlazza M. J.; Bilinovich S.; Prokop J. W.; Hill A. L.; Williams D. C. Methylation Specific Targeting of a Chromatin Remodeling Complex from Sponges to Humans. Sci. Rep. 2017, 7, 40674 10.1038/srep40674. 28094816 PMC5240623

[ref27] Sperlazza M. J.; Bilinovich S. M.; Sinanan L. M.; Javier F. R.; Williams D. C. Structural Basis of MeCP2 Distribution on Non-CpG Methylated and Hydroxymethylated DNA. J. Mol. Biol. 2017, 429, 1581–1594. 10.1016/j.jmb.2017.04.009. 28450074 PMC5492995

[ref28] Cavanagh J.; Fairbrother W. J.; Palmer A. G. III; Rance M.; Skelton N. J. Protein NMR Spectroscopy, 2nd Ed.; Elsevier, 2007.

[ref29] Kleckner I. R.; Foster M. P. An Introduction to NMR-Based Approaches for Measuring Protein Dynamics. Biochim. Biophys. Acta 2011, 1814, 942–968. 10.1016/j.bbapap.2010.10.012. 21059410 PMC3061256

[ref30] Hashimoto H.; Liu Y.; Upadhyay A. K.; Chang Y.; Howerton S. B.; Vertino P. M.; Zhang X.; Cheng X. Recognition and Potential Mechanisms for Replication and Erasure of Cytosine Hydroxymethylation. Nucleic Acids Res. 2012, 40, 4841–4849. 10.1093/nar/gks155. 22362737 PMC3367191

[ref31] Scarsdale J. N.; Webb H. D.; Ginder G. D.; Williams D. C. Solution Structure and Dynamic Analysis of Chicken MBD2 Methyl Binding Domain Bound to a Target-Methylated DNA Sequence. Nucleic Acids Res. 2011, 39, 6741–6752. 10.1093/nar/gkr262. 21531701 PMC3159451

[ref32] Zemach A.; Grafi G. Characterization of Arabidopsis Thaliana Methyl-CpG-Binding Domain (MBD) Proteins. Plant J. 2003, 34, 565–572. 10.1046/j.1365-313x.2003.01756.x. 12787239

[ref33] Berg A.; Meza T. J.; Mahić M.; Thorstensen T.; Kristiansen K.; Aalen R. B. Ten Members of the Arabidopsis Gene Family Encoding Methyl-CpG-Binding Domain Proteins Are Transcriptionally Active and at Least One, AtMBD11, Is Crucial for Normal Development. Nucleic Acids Res. 2003, 31, 5291–5304. 10.1093/nar/gkg735. 12954765 PMC203319

[ref34] Scebba F.; Bernacchia G.; De Bastiani M.; Evangelista M.; Cantoni R. M.; Cella R.; Locci M. T.; Pitto L. Arabidopsis MBD Proteins Show Different Binding Specificities and Nuclear Localization. Plant Mol. Biol. 2003, 53, 755–771. 10.1023/B:PLAN.0000019118.56822.a9. 15010609

[ref35] Ito M.; Koike A.; Koizumi N.; Sano H. Methylated DNA-Binding Proteins from Arabidopsis. Plant Physiol. 2003, 133, 1747–1754. 10.1104/pp.103.026708. 14605234 PMC300729

[ref36] Johnson L. M.; Bostick M.; Zhang X.; Kraft E.; Henderson I.; Callis J.; Jacobsen S. E. The SRA Methyl-Cytosine-Binding Domain Links DNA and Histone Methylation. Curr. Biol. 2007, 17, 379–384. 10.1016/j.cub.2007.01.009. 17239600 PMC1850948

[ref37] Rajakumara E.; Law J. A.; Simanshu D. K.; Voigt P.; Johnson L. M.; Reinberg D.; Patel D. J.; Jacobsen S. E. A Dual Flip-out Mechanism for 5mC Recognition by the Arabidopsis SUVH5 SRA Domain and Its Impact on DNA Methylation and H3K9 Dimethylation in Vivo. Genes Dev. 2011, 25, 137–152. 10.1101/gad.1980311. 21245167 PMC3022260

[ref38] Du J.; Johnson L. M.; Groth M.; Feng S.; Hale C. J.; Li S.; Vashisht A. A.; Gallego-Bartolome J.; Wohlschlegel J. A.; Patel D. J.; Jacobsen S. E. Mechanism of DNA Methylation-Directed Histone Methylation by KRYPTONITE. Mol. Cell 2014, 55, 495–504. 10.1016/j.molcel.2014.06.009. 25018018 PMC4127122

[ref39] Brzeski J.; Jerzmanowski A. Deficient in DNA Methylation 1 (DDM1) Defines a Novel Family of Chromatin-Remodeling Factors. J. Biol. Chem. 2003, 278, 823–828. 10.1074/jbc.M209260200. 12403775

[ref40] Markley J. L.; Bax A.; Arata Y.; Hilbers C. W.; Kaptein R.; Sykes B. D.; Wright P. E.; Wüthrich K. Recommendations for the Presentation of NMR Structures of Proteins and Nucleic Acids. Pure Appl. Chem. 1998, 70, 117–142. 10.1351/pac199870010117. 9671561

[ref41] Delaglio F.; Grzesiek S.; Vuister G.; Zhu G.; Pfeifer J.; Bax A. NMRPipe: A Multidimensional Spectral Processing System Based on UNIX Pipes. J. Biomol. NMR 1995, 6, 277–293. 10.1007/BF00197809. 8520220

[ref42] Vranken W. F.; Boucher W.; Stevens T. J.; Fogh R. H.; Pajon A.; Llinas M.; Ulrich E. L.; Markley J. L.; Ionides J.; Laue E. D. The CCPN Data Model for NMR Spectroscopy: Development of a Software Pipeline. Proteins 2005, 59, 687–696. 10.1002/prot.20449. 15815974

[ref43] Kobayashi N.; Iwahara J.; Koshiba S.; Tomizawa T.; Tochio N.; Güntert P.; Kigawa T.; Yokoyama S. KUJIRA, a Package of Integrated Modules for Systematic and Interactive Analysis of NMR Data Directed to High-Throughput NMR Structure Studies. J. Biomol. NMR 2007, 39, 31–52. 10.1007/s10858-007-9175-5. 17636449

[ref44] Johnson B. A.; Blevins R. A. NMR View: A Computer Program for the Visualization and Analysis of NMR Data. J. Biomol. NMR 1994, 4, 603–614. 10.1007/BF00404272. 22911360

[ref45] Rückert M.; Otting G. Alignment of Biological Macromolecules in Novel Nonionic Liquid Crystalline Media for NMR Experiments. J. Am. Chem. Soc. 2000, 122, 7793–7797. 10.1021/ja001068h.

[ref46] Downing A. K. Protein NMR Techniques, 2nd ed.; Humana Press, 2004; Vol. 278.

[ref47] Shen Y.; Delaglio F.; Cornilescu G.; Bax A. TALOS+: A Hybrid Method for Predicting Protein Backbone Torsion Angles from NMR Chemical Shifts. J. Biomol. NMR 2009, 44, 213–223. 10.1007/s10858-009-9333-z. 19548092 PMC2726990

[ref48] Schwieters C. D.; Kuszewski J. J.; Tjandra N.; Marius Clore G. The Xplor-NIH NMR Molecular Structure Determination Package. J. Magn. Reson. 2003, 160, 65–73. 10.1016/s1090-7807(02)00014-9. 12565051

[ref49] Schwieters C.; Kuszewski J.; Mariusclore G. Using Xplor–NIH for NMR Molecular Structure Determination. Prog. Nucl. Magn. Reson. Spectrosc. 2006, 48, 47–62. 10.1016/j.pnmrs.2005.10.001.

[ref50] Sattler M.; Schleucher J.; Griesinger C. Heteronuclear Multidimensional NMR Experiments for the Structure Determination of Proteins in Solution Employing Pulsed Field Gradients. Prog. Nucl. Magn. Reson. Spectrosc. 1999, 34, 93–158. 10.1016/S0079-6565(98)00025-9.

[ref51] Schmidt E.; Güntert P. A New Algorithm for Reliable and General NMR Resonance Assignment. J. Am. Chem. Soc. 2012, 134, 12817–12829. 10.1021/ja305091n. 22794163

[ref52] Marsh J. A.; Singh V. K.; Jia Z.; Forman-Kay J. D. Sensitivity of Secondary Structure Propensities to Sequence Differences between α- and γ-Synuclein: Implications for Fibrillation. Protein Sci. 2006, 15, 2795–2804. 10.1110/ps.062465306. 17088319 PMC2242444

[ref53] Williamson M. P. Using Chemical Shift Perturbation to Characterise Ligand Binding. Prog. Nucl. Magn. Reson. Spectrosc. 2013, 73, 1–16. 10.1016/j.pnmrs.2013.02.001. 23962882

[ref54] Sugase K.; Konuma T.; Lansing J. C.; Wright P. E. Fast and Accurate Fitting of Relaxation Dispersion Data Using the Flexible Software Package GLOVE. J. Biomol. NMR 2013, 56, 275–283. 10.1007/s10858-013-9747-5. 23754491 PMC3735449

