# Supplementary material for: Structural Insights into Methylated DNA Recognition by the Methyl-CpG Binding Domain of MBD6 from Arabidopsis thaliana
Source: ACS Omega. 2022 Jan 19;7(4):3212–21. doi: 10.1021/acsomega.1c04917 (PMC8811898; doi:10.1021/acsomega.1c04917)
Supplement: Supplementary file 1 — ao1c04917_si_001.pdf [file ao1c04917_si_001.pdf]

Supporting Information

Structural insights into methylated DNA recognition by the methyl-CpG binding domain of MBD6 from *Arabidopsis thaliana*

Yutaka Mahana[1], Izuru Ohki[2], Erik Walinda[3], Daichi Morimoto[1], Kenji Sugase[1], and Masahiro Shirakawa[1]*

[1]Department of Molecular Engineering, Kyoto University, Kyoto-Daigaku Katsura, Nishikyo-Ku, Kyoto 615-8510, Japan

[2]Institute for Chemical Research, Kyoto University, Gokasho, Uji, Kyoto 611-0011, Japan

[3]Graduate School of Medicine, Kyoto University, Yoshida Konoe-Cho, Sakyo-Ku, Kyoto 606-8501, Japan

This file includes Supporting Figures S1–S6.

Supporting Figures

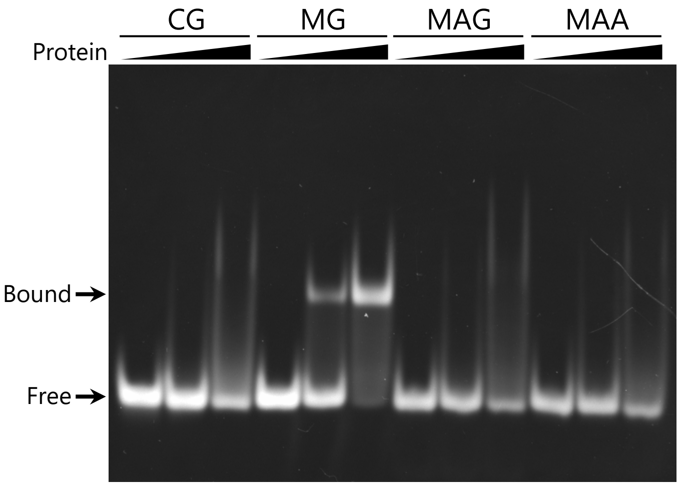

Figure S1. Gel shift assay of DNA binding by the AtMBD6 MBD domain at different concentrations. The molar ratios of the protein to DNA in the reactions were 0, 1, 5 for the left, middle, and right lanes, respectively.

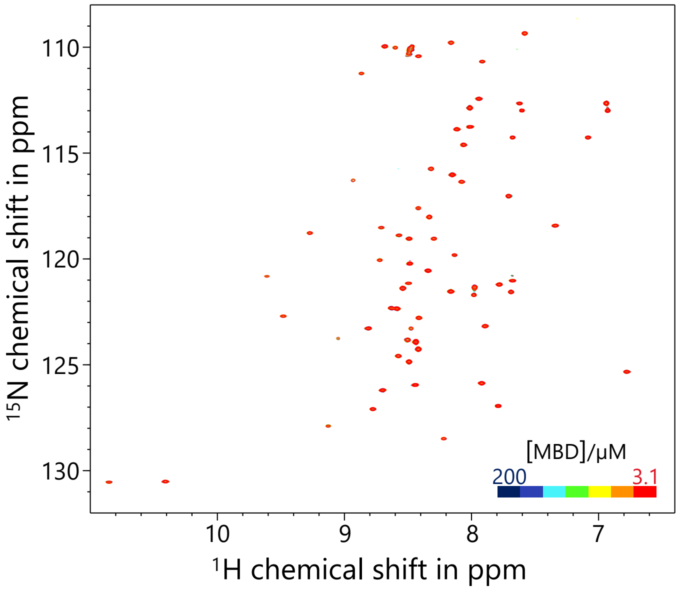

Figure S2. Comparison of backbone amide chemical shifts of the AtMBD6 MBD domain at different concentrations. The virtually identical spectra demonstrate that MBD_{AtMBD6} exists as a monomer and is not in a monomer-homodimer equilibrium at micromolar concentrations.

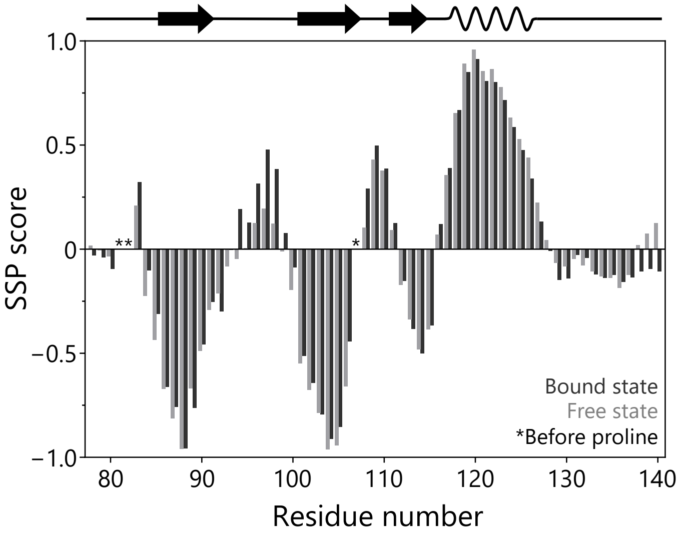

Figure S3. Comparison of the SSP scores of the AtMBD6 MBD domain in the free and bound states. The SSP scores of MBD_{AtMBD6} in the bound state (black) were calculated by the SSP program. For comparison, the SSP scores in the free state (gray) estimated in our previous study[21] are displayed. Schematic representation of the secondary structure is shown at the top.

```
                                     R    R       R
                                     K    RK      K D Y            R                        FBF
AtMBD1    107-183 SRTWVIDKPGLPRTPRGFKRSLILRKDY---SKMDAYY--ITP-TGKKLKSRNEIAAFIDANQDYK--YALLGDFNFTVPKVMEE
AtMBD2    115-195 TRLWAIDKPNISRPPAGWQRLLRIRGEGG-TRFADVYY--VAP-SGKKLRSTVEVQKYLNDNSEYIGEGVKLSQFSFQIPKPLQD
AtMBD3     62-140 TMRWFQDEHSIPKTPQGLKRVLVVRTNC---VKVDVYYESLAP-RRKRFKSIKEVATFIEDKEEFK--DMTLEEVSFAAPKRLKL
AtMBD4     80-156 SRTWVIDKPGLPKTPKGFKRSLVLRKDY---SKMDTYY--FTP-TGKKLRSRNEIAAFVEANPEFR--NAPLGDFNFTVPKVMED
AtMBD5     22-104 KSRKRATPGDDNWLPPDWRTEIRVRTSGTKAGTVDKFY--YEPITGRKFRSKNEVLYYLEHGTPKKKSVKTAENGDSHSEHSEGR
AtMBD6     69-149 KSRKRAAPGD-NWLPPGWRVEDKIRTSGATAGSVDKYY--YEPNTGRKFRSRTEVLYYLEHGTSKR-GTKKAENTYFNPDHFEGQ
AtMBD7-a    18-98 QLQIADPTSFCGKIMPGWTVVNRPRSSN--NGVVDTYF--IEPGTGRQFSSLEAIHRHLAGEVNDRRLTRAGSFFQDKTRVYEGS
AtMBD7-b  103-168 DHCGVEYASKGFRLPRGWSVEEVPRKN---SHYIDKYY--VERKTGKRFRSLVSVERYLRESRNSIEQQLR--------------
AtMBD7-c  169-248 VLQNRRGHSKDFRLPDGWIVEEKPRRS---SSHIDRSY--IEPGTGNKFRSMAAVERYLISVGNITLDSVSMVHSERLPLLMNRN
AtMBD8    331-412 KKKVVNACDYGGYLPRGWRLMLYIKRKGSNLLLACRRY--ISP-DGQQFETCKEVSTYLRSLLESPSKNQHYYLQSDNKTLGQQP
AtMBD9    255-333 QNLRHFISERHGVLEDGWRVEFRQPLNG---YQLCAVY--CAP-NGKTFSSIQEVACYLGLAINGNYSCMDAEIRNENSLLQERL
AtMBD10      1-77 MENTDELVSIELPAPASWKKLFYPKRAG-TPRKTEIVF--VAP-TGEEISSRKQLEQYLKAHPGNP----VISEFEWTTGETPRR
AtMBD11      1-77 MGGEEEVVSVELPAPSSWKKLFYPNKVG-SVKKTEVVF--VAP-TGEEISNRKQLEQYLKSHPGNP----AIAEFDWTTSGTPRR
AtMBD12    50-129 DGTTCDTWPSIPPIPTGWSRSVHIRSES--TKFADVYY--FPP-SGERLRSSAEVQSFLDNHPEYVREGVNRSQFSFQIPKPLDD
AtIDM1    239-318 PRPLLYKYVCKVLTAARWKIEKRERSAG--RKHVDTFY--ISP-EGRKFREFGSAWKALGGILLADRKLMDTGTKKWTGINDFWS
```

Figure S4. Sequence alignment of the MBD domains in *A. thaliana*. Identical and homologous residues are colored in black and gray, respectively. The important residues for specific recognition of the methyl-CpG site and formation of the hairpin loop motif conserved among mammalian MBD domains are indicated on the top.

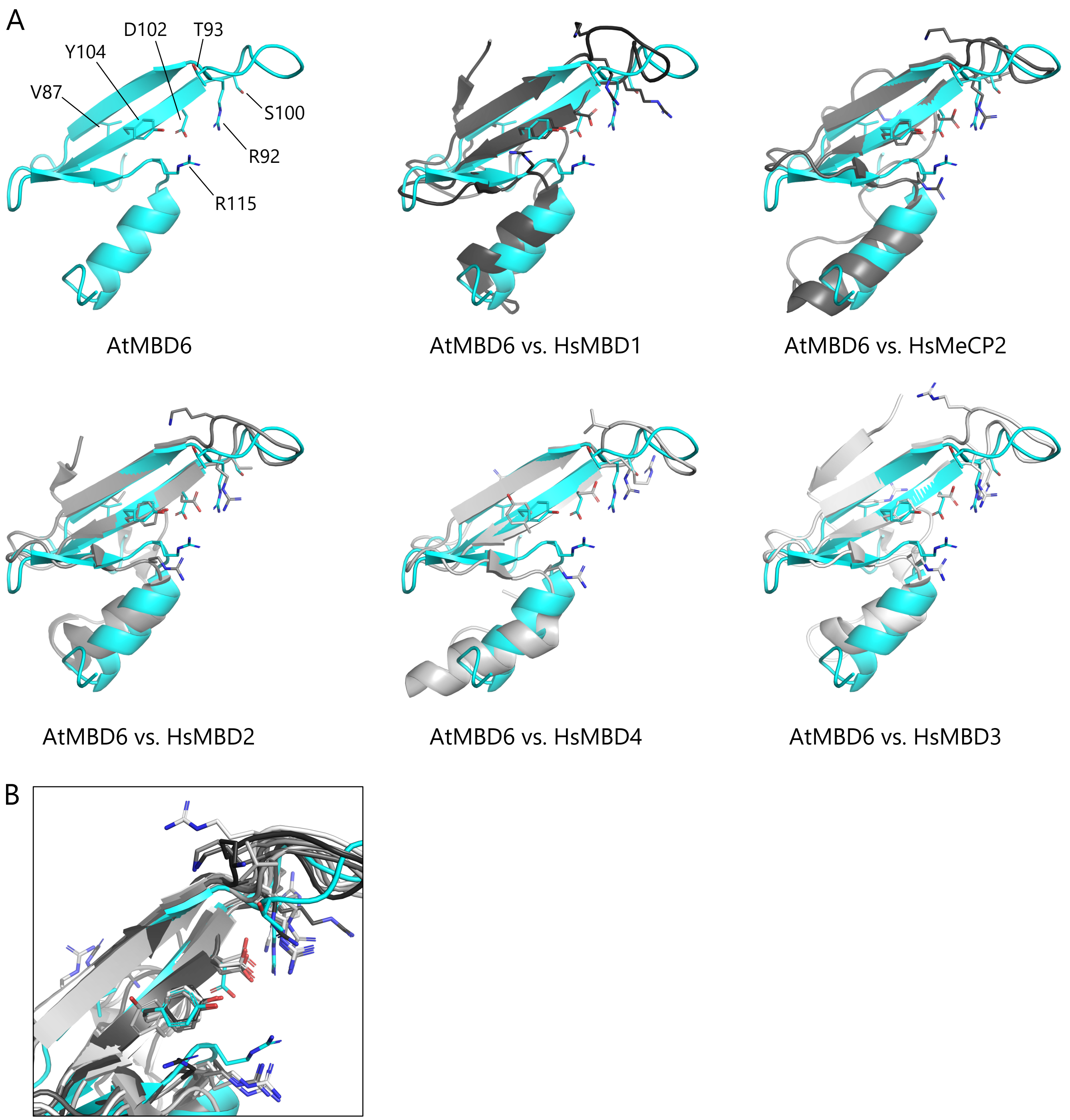

Figure S5. Structural superposition of the AtMBD6 MBD domain and human MBD domains. (A) Superposition of the solution structure of MBD_{AtMBD6} in the free form (cyan) and the crystal or solution structure of human MBD domains bound to methyl-CpG-containing DNA (gray). DNA moieties are omitted for clarity. Side-chains of important residues marked by asterisks and daggers in Figure 4A are shown as sticks. Protein Data Bank IDs of the human MBD domains shown are: HsMBD1, 1IG4; HsMeCP2, 3C2I; HsMBD2, 6CNQ; HsMBD4, 2MOE; HsMBD3, 6CCG. (B) Superposition of all six MBD domains shown in Figure S5A focusing on the important residues.

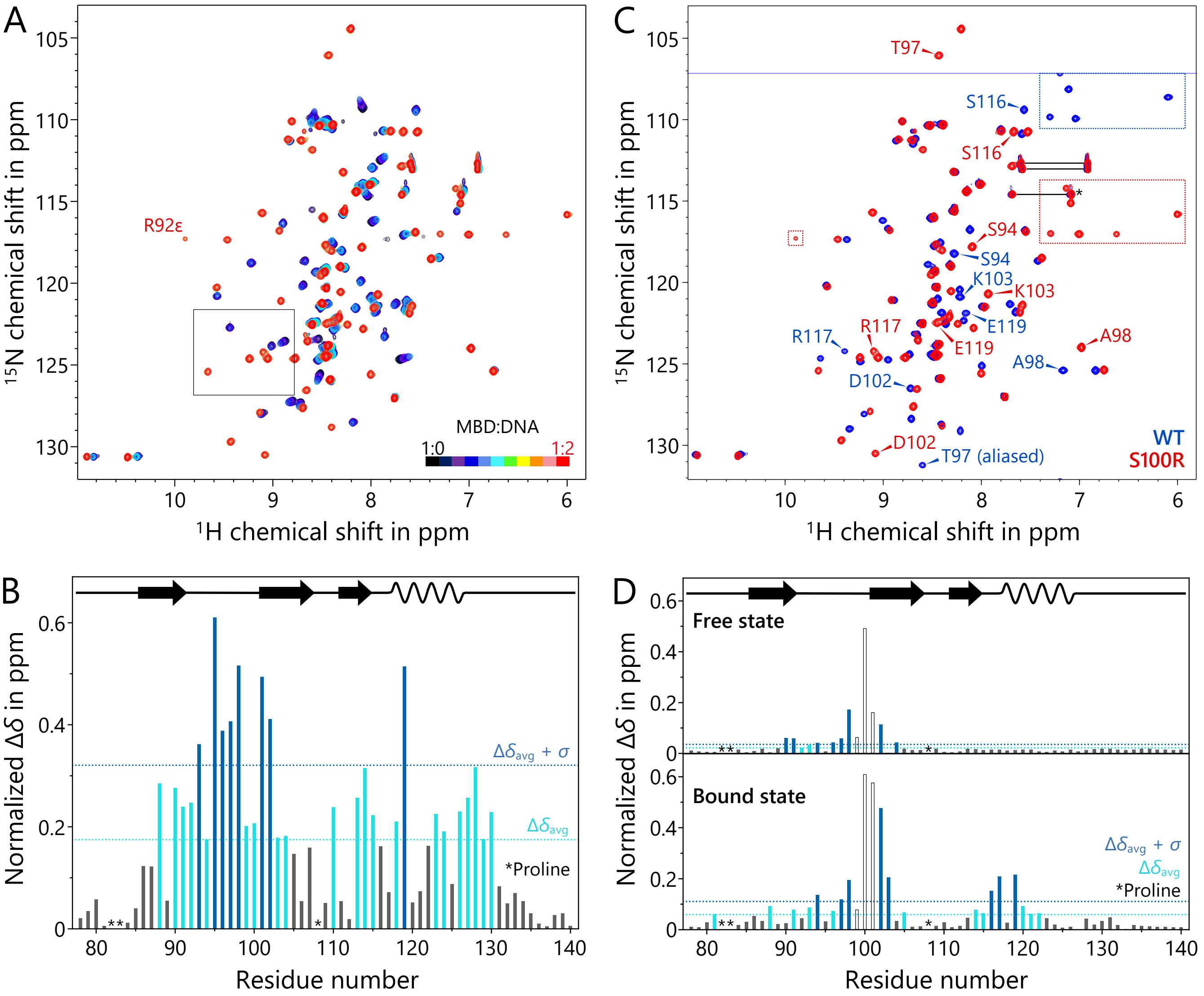

Figure S6. Binding of the S100R AtMBD6 MBD domain to methyl-CpG-containing DNA. (A) Overall view of the spectral changes of S100R MBD_{AtMBD6} over the course of the titration. The enclosed region corresponds to Figure 4D. (B) Normalized CSD values of S100R MBD_{AtMBD6} upon binding toward methyl-CpG-containing DNA. (C) Spectral overlay of WT (blue) and S100R (red) MBD_{AtMBD6} bound to methyl-CpG-containing DNA (the molar ratios of the protein to DNA are 1:1.5 and 1:2, respectively). The blue line indicates the spectral border in the ^{15}N dimension of the spectrum of WT MBD_{AtMBD6}. For clarity, only residues showing large CSD values between WT and S100R MBD_{AtMBD6} (as marked in blue in Figure S6D, bottom) are annotated. The paired side-chain amide resonances of asparagine and glutamine residues are connected by black horizontal lines. Cross-peaks in the areas surrounded by dashed lines, except for an amide NH_2 signal marked with an asterisk, correspond to aliased side-chain resonances. (D) Comparison of backbone amide chemical shifts between WT MBD_{AtMBD6} and the S100R mutant. Residues 99–101 were excluded in the calculation of the average values and the standard deviations.
